# Polypharmacy is associated with malnutrition and activities of daily living disability among daycare facility users: A cross-sectional study

**DOI:** 10.1097/MD.0000000000027073

**Published:** 2021-08-27

**Authors:** Tomiyo Nakamura, Takashi Itoh, Aiko Yabe, Shoko Imai, Yoshimi Nakamura, Yasuko Mizokami, Yuki Okouchi, Akito Ikeshita, Hidenori Kominato

**Affiliations:** a Department of Food Sciences and Human Nutrition, Ryukoku University, Shiga; b Department of Molecular Pathology, Osaka University Graduate School of Medicine & Health Science, Osaka; c Pharmarise Co., Ltd., Tokyo; d I&H Co., Ltd., Hyogo, Japan.

**Keywords:** activities of daily living, anorexia, daycare facility, malnutrition, polypharmacy, proton pump inhibitor

## Abstract

Polypharmacy influences malnutrition and activities of daily living (ADL) in older individuals owing to side effects such as anorexia. This study aimed to examine whether polypharmacy (5 or more drugs) is associated with malnutrition and ADL disability among daycare facility users.

This cross-sectional study was performed in a daycare facility specializing in rehabilitation. Malnutrition was defined according to the Global Leadership Initiative on Malnutrition criteria and ADL disability according to the “criteria for determination of the daily life independence level (bedridden level) of elderly with disabilities.”

In total, 103 of the 134 included individuals were analyzed. Thirty-three (32.0%) participants were malnourished, 46 (44.7%) had ADL disability, 58 (56.3%) qualified as cases of polypharmacy, and 9 (8.7%) experienced loss of appetite. Multivariable logistic regression analysis showed that polypharmacy was independently associated with malnutrition and ADL disability. Separate analyses of each type of drug revealed that proton pump inhibitors (that impair protein absorption and assimilation), anticonstipation drugs, and antihypertensive drugs were associated with malnutrition, whereas proton pump inhibitors, anticonstipation drugs, antidyslipidemia drugs, and antidiabetic drugs were associated with ADL disability. The only factor related to anorexia was the loss of pleasure of eating, which in turn was related to psychological stress.

The side effects of polypharmacy among individuals with malnutrition and ADL disability may include impaired protein absorption and assimilation caused by proton pump inhibitors, but not anorexia. Further multicenter prospective studies are required to confirm these findings.

## 1 Introduction

In 2020, the number of older people in Japan reached a record high of 36.17 million (28.7% of the total population), making it the country with the highest proportion of older people worldwide.^[1]^ This aging trend has been accompanied by an increase in the number of people who require long-term care and assistance.^[2]^ The number of older people living at home and using daycare facilities to prevent the need for long-term care continues to rise annually; the current number is estimated at 2.26 million, comprising approximately 1 in 3 people.^[3]^

The prevalence of frailty among community-dwelling older people in Japan is 8.7%, and the risk of frailty has been reported to be 40.8%.^[4]^ Malnutrition is at the core of the frailty cycle, and the loss of muscle mass, reduced muscle strength, weakness, and reduced energy expenditure may trigger a vicious cycle of anorexia.^[5]^ According to the definition used by Japan's long-term care insurance system, approximately 25% of those who require long-term care have problems related to locomotive organ dysfunction and deterioration.^[6]^ Malnutrition among users of daycare facilities is associated with activities of daily living (ADL) disability, reduced quality of life, a compromised immune system, infections, and the need for prolonged treatment, all of which have an effect on the vital prognosis.^[7]^

Approximately 40% to 60% of daycare facility users are malnourished.^[8,9]^ The prevalence rate differs according to the definition of malnutrition. When the Mini Nutritional Assessment-Short Form (MNA-SF)^[10,11]^ was employed, the malnutrition prevalence was 60%; however, using the 2018 Global Leadership Initiative on Malnutrition (GLIM) criteria,^[12]^ the proportion was 40%.^[9]^ Sanchez-Rodriguez et al^[13]^ reported that over the 4-year follow-up period, malnutrition defined according to the GLIM criteria was associated with a 4.4 times higher risk of death, and defined according to the European Society for Clinical Nutrition and Metabolism criteria^[14]^ was associated with a 2.2 times higher risk of death than in the general population. The use of the GLIM criteria allows for a more appropriate identification of malnutrition, which suggests that these criteria may be more helpful than others for providing care to community-dwelling older people. Thus, the urgent task we are faced with is the identification of malnutrition among community-dwelling older people and the implementation of preventative measures.

Although multiple factors are related to malnutrition among older individuals, evidence suggests that anorexia during aging may be an important indicator of malnutrition.^[15]^ A systematic review of observational studies concluded that anorexia is strongly related to malnutrition in community-dwelling older people, and that diabetes, hospitalization, self-reported poor health status, and lack of teeth are moderately related.^[16]^ A systematic review of prospective cohort studies found moderate evidence that hospitalization, food dependence, self-recognized poor health status, decreased physical function, and anorexia are determining factors for malnutrition.^[17]^ A scoping review identified anorexia as an obstacle to food intake among participants in the majority of the included studies.^[18]^

The factors associated with anorexia-related malnutrition include drug side effects.^[19,20]^ Depending on the specific type of drug administered, side effects may include anorexia, constipation, diarrhea, and depression, all of which induce malnutrition.^[21]^ In addition to the type of drug, the concurrent use of multiple drugs may adversely affect nutritional status.^[22]^ The frequency of malnutrition and polypharmacy (the concurrent use of 5 or more drugs) increases with age, and these conditions have a major impact on the quality of life and mortality rate among older people.^[23]^ Moreover, polypharmacy is a major factor associated with frailty in older individuals.^[24]^

Given that older people are likely to have a variety of concurrent chronic ailments, they are prone to polypharmacy. In Japan, a single patient aged 75 years or older is able to fill prescriptions for 4.6 drugs per month, and many (40.3%) older people fill prescriptions for five or more types of drugs.^[25]^ Approximately 5% of older people who are hospitalized on an emergency basis experience adverse drug effects (ADEs), which is significantly related to polypharmacy.^[26]^ Patients who use antiplatelet drugs and anticoagulants often experience ADE, the most common being gastrointestinal bleeding and nausea, which are both related to anorexia. In addition, 34.0% of older people who were beneficiaries of regular home visit services were found to be using potentially inappropriate drugs.^[27]^

However, the effect of polypharmacy on malnutrition and ADL disability among daycare facility users is unknown. Thus, the aim of this study was to elucidate the relationship between malnutrition, ADL disability, and polypharmacy among daycare facility users.

## 2 Methods

### 2.1 Participants

This cross-sectional study was performed in a single daycare facility specializing in rehabilitation. The exclusion criteria were as follows:

(1) Users who did not provide consent to participate in this study;
(2) Users who exhibited a marked decline in cognitive function and a loss of decision-making abilities;
(3) Users determined to be inappropriate for this study by the principal investigator.

The participants were recruited in November 2018, and the survey period extended from December 3 to December 12 of the same year (a total of 8 weekdays).

The study participants were provided with written and oral descriptions of the objectives and details of the survey. Only participants who provided consent to participate and for the purpose of publication were included in the study. This study was conducted in accordance with the principles of the Declaration of Helsinki, and the study protocol was reviewed and approved by the institutional review board for studies involving human subjects associated with Ryukoku University (approval no. 2018–19).

### 2.2 Data collection

The survey included the following 5 items: (1) physical measurements, (2) basic clinical characteristics of the participants, (3) responses to the MNA-SF survey, (4) answers to a questionnaire survey (subjective symptoms along with living and dietary habits), and (5) drug intake status.

(1) Physical measurements: Height and weight measurements were performed by the facility staff and 4 registered dietitians, and calf circumference (CC) measurements were performed by 4 registered dietitians. Training was conducted before measurements to ensure accuracy. Body mass index (BMI) was calculated by dividing the weight (kg) by the square of height (m^2^). CC was used as an indicator of decreased muscle mass.

(2) Basic clinical characteristics of the participants: We collected data on the following basic clinical characteristics of the participants: sex, date of birth, history of present illness or medical history, ambulatory status, frequency of facility usage, level of long-term care required according to the Japanese long-term care insurance system,^[28]^ criteria for determination of the daily life independence level (bedridden level) of the elderly with disability,^[29]^ and criteria for determination of the daily life independence level of the elderly with dementia (rating of dementia).^[30]^ These data were obtained from the basic information records of the facility office.

The levels of long-term care required were defined as requiring assistance (grades 1 and 2) and requiring long-term care (grades 3–5). Forty-eight (46.6%) participants qualified as “requiring assistance” (grade 1 or 2) and 20 (19.4%) participants qualified as “requiring long-term care” (grades 3 or 4). None of the participants qualified as requiring grade 5 assistance. Hence, participants who required grade 1 or 2 assistance and those who required grade 3 or 4 assistance were dichotomized into the following categories: those requiring assistance and those requiring long-term care. The item “bedridden level” was divided into “independent in daily life,” “at risk of becoming bedridden” (grade A), and “bedridden” (grades B or C).^[31]^ Only 2 participants were classified as “bedridden”; therefore, those who were classified as “independent in daily life” were included in the “independent” category, and the remaining participants were included in the “ADL disability” category. Although the “rating of dementia” includes the categories of “independent” (grade I), “mild dementia” (grade II [IIa & IIb]), “moderate dementia” (grade III [IIIa & IIIb]), “severe dementia” (grade IV), and “severe dementia, required specialized medical care” (grade M), the study participants were classified as either “independent” or having “mild dementia.” The presence of chronic diseases was determined through interviews with patients by examining the patients’ drug intake status and through interviews with the director and the nurse of the facility.

(3) Responses to the MNA-SF and (4) Answers to the questionnaire survey: These were obtained through interviews with 4 registered dietitians. Psychological stress was scored using the responses of either “yes” or “no.” Neuropsychological problems were defined as no psychological problems (coded as 0), depression, or mild dementia (coded as 1). The frequency of meal intake was determined through interviews in which the participants were asked about the frequency of their weekly consumption of proteins including meat, seafood, eggs, soybeans and related foods, and milk and dairy products. Participants who reported consuming at least 3 sources of protein per day were placed in the “≥3 a day” group, and those who reported consuming fewer than 3 sources per day were placed in the “< 3 a day” group.

Anorexia is characterized by abnormal loss of appetite. Responses to questions on appetite were categorized as either “healthy appetite” or “loss of appetite.” Responses to the question, “Is eating enjoyable?” were handled as follows: participants who responded “yes” were listed as “yes” in the “pleasure of eating” category, and those who responded “no” were listed as “loss of pleasure of eating” in the same category. Responses to the question, “Are you eating together at least once per day?” were categorized as either “yes” or “no.”

(5) Drug intake status: Drug intake status was determined through interviews and by examining drug prescription records provided by the participants. Currently, there is no clear definition of polypharmacy based on the number of drugs used.^[19]^ In this study, “polypharmacy” was defined as using 5 or more drugs concurrently. The types of drugs used were determined by a pharmacist and categorized as follows: antihypertensive drugs, proton pump inhibitors (PPIs), anticonstipation drugs, antiplatelet drugs, antidyslipidemia drugs, sleeping aids, antidiabetic drugs, anticoagulants, probiotics, and gastrointestinal motility regulators.

Malnutrition was diagnosed based on GLIM criteria. MNA-SF was used as a nutritional screening tool.

### 2.3 Sample size

The prevalence of malnutrition and ADL disability among daycare facility users, the outcome of this study, was expected to be approximately 50%.^[8,9,32]^ We set the precision of the estimate at 10% and the Z-value at 1.96. A total sample size of 101 was calculated, considering a loss-to-follow-up rate of 5%.

### 2.4 Statistical analysis

All statistical analyses were performed using IBM SPSS Statistics for Windows, version 27.0, (IBM Corp., Armonk, NY). To reduce the amount of missing data, we obtained these data from the participants 1 week after surgery. Eighty-one (78.6%) participants did not have a past weight record from which to calculate weight loss; we elicited their weight history whenever possible by asking the nurse about their weight loss. Three participants who reported that they did not know the frequency of consumption of any food were considered not to eat. Categorical data, expressed as number (percentage) of participants, were compared using the Chi-square test. When the expected values were ≤5, Fisher's exact test was used. Continuous values are shown as the mean ± standard deviation (SD) and included normalized and nonparametric values. Nonparametric variables, including age, MNA-SF scores, and daily consumption of high-protein foods, were compared using the Mann–Whitney *U* test. BMI and CC results were compared using independent sample *t* tests. Participants were divided according to the GLIM criteria as “well-nourished” and “malnourished,” bedridden level was categorized as “independent” and “ADL disability,” and appetite was classified as either “healthy appetite” or “loss of appetite.” When these factors were found to be significant in the univariate analysis, they were considered as confounding factors and were used as covariates in the multivariable logistic regression analysis. Statistical significance was set at *P* < .05.

## 3 Results

**Figure 1:**
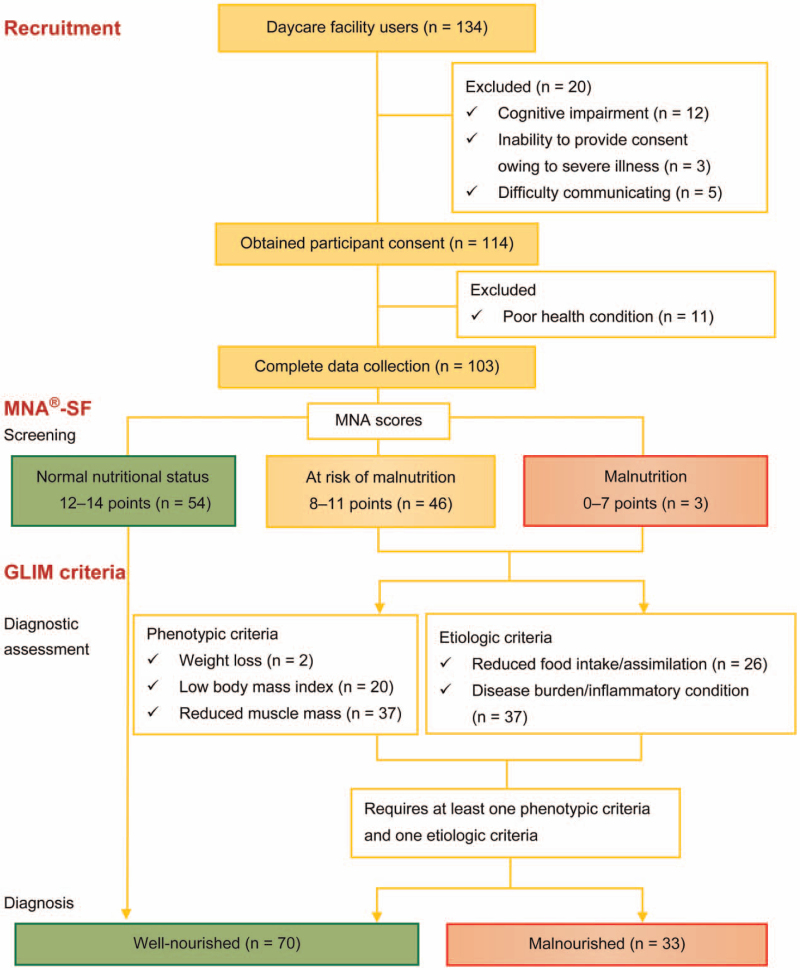
Diagnostic algorithm for malnutrition. GLIM = Global Leadership Initiative on Malnutrition, MNA-SF = Responses to the Mini Nutritional Assessment-Short Form.

Twenty of the 134 participants enrolled in this study were excluded during the study period (Fig. 1). The reasons for exclusion were as follows: 12 participants had decreased cognitive function, 3 were unable to provide consent owing to severe illness, and 5 were unable to provide consent owing to their inability to communicate. Although consent to participate was obtained from 114 (85.1%) participants, 11 were unable to participate because of poor physical condition, including acute disease. Hence, 103 participants (8 people with disabilities and 95 older people) were included in this study. Forty-nine (47.6%) of the 103 participants who underwent MNA-SF screening were found to have malnutrition or were at risk of malnutrition. A diagnostic assessment based on the phenotypic and etiologic components of the GLIM criteria indicated that 70 (68.0%) participants were well-nourished and 33 (32.0%) were malnourished.

The characteristics of the study participants are listed in Table 1. The mean age was 79 years (range, 47–64 years). The polypharmacy rate was 47.1% in the well-nourished group and 75.8% in the malnourished group, indicating that the polypharmacy rate in the malnourished group was significantly higher (*P* = .005). In addition, the malnourished group had a significantly lower BMI and CC, but significantly higher rates of ADL disability, chronic diseases, mild dementia, and neuropsychological problems than the well-nourished group. Nearly all participants (91.3%) reported that their appetite was good, which suggests that they maintained a healthy appetite. No significant differences were found between groups.

**Table 1:** Characteristics of the study participants.

		GLIM criteria, n (%)		
Variable	All (n = 103)	Well-nourished (n = 70)	Malnourished (n = 33)	*P*
Age, yr	79.0 ± 8.8	78.8 ± 8.5	79.3 ± 9.5	.60
≤74	22 (21.4)	15 (21.4)	7 (21.2)	.60
≥75	81 (78.6)	55 (78.6)	26 (78.8)	
Sex				
Male	52 (50.5)	34 (48.6)	18 (54.5)	.36
Female	51 (49.5)	36 (51.4)	15 (45.5)	
BMI, kg/m^2^	23.2 ± 3.9	24.4 ± 3.8	20.6 ± 2.8	<.01
≥18.5 if <70 yr, ≥20 if ≥70 yr	81 (78.6)	63 (90.0)	18 (54.5)	<.01
<18.5 if <70 yr, <20 if ≥70 yr	22 (21.4)	7 (10.0)	15 (45.5)	
CC, cm	32.9 ± 3.4	34.0 ± 3.1	30.5 ± 2.5	<.01
≥34 for male, ≥33 for female	47 (45.6)	45 (64.3)	2 (6.1)	<.01
<34 for male, <33 for female	56 (54.4)	25 (35.7)	31 (93.9)	
MNA-SF	11.4 ± 2.1	12.3 ± 1.7	9.5 ± 1.3	<.01
Normal	54 (52.4)	54 (77.1)	…	
Malnourished/at risk of malnutrition	49 (47.6)	16 (22.9)	33 (100.0)	…
High protein-source foods	2.77 ± 1.33	2.77 ± 1.40	2.74 ± 1.16	.76
<3 per day	66 (64.1)	45 (64.3)	21 (63.6)	.56
≥3 per day	37 (35.9)	25 (35.7)	12 (36.4)	
Nursing care level				
Requiring assistance	35 (34.0)	27 (38.6)	8 (24.2)	.11
Long-term care level	68 (66.0)	43 (61.4)	25 (75.8)	
Bedridden level				
Independent	57 (55.3)	43 (61.4)	14 (42.4)	.06
ADL disability	46 (44.7)	27 (38.6)	19 (57.6)	
Rating of dementia				
Independent	76 (73.8)	57 (80.0)	20 (60.6)	.03
Mild dementia	27 (26.2)	14 (20.0)	13 (39.4)	
Chronic diseases				
Yes	69 (67.0)	37 (52.9)	32 (97.0)	<.01
No	34 (33.0)	33 (47.1)	1 (3.0)	
Polypharmacy				
<5 types of drugs	45 (43.7)	37 (52.9)	8 (24.2)	<.01
≥5 types of drugs	58 (56.3)	33 (47.1)	25 (75.8)	
Neuropsychological problems				
Yes	20 (19.4)	9 (12.9)	11 (33.3)	.01
No	83 (87.1)	61 (87.1)	22 (66.7)	
Psychological stress				
Yes	32 (31.1)	13 (18.6)	19 (57.6)	<.01
No	71 (68.9)	57 (81.4)	14 (42.4)	
Appetite				
Healthy appetite	94 (91.3)	65 (92.9)	29 (87.9)	.31
Loss of appetite	9 (8.7)	5 (7.1)	4 (12.1)	
Pleasure of eating				
Pleasure of eating	85 (82.5)	63 (90.0)	22 (66.7)	<.01
Loss of pleasure of eating	18 (17.5)	7 (10.0)	11 (33.3)	
Eating together at least once per day				
Yes	71 (68.9)	47 (67.1)	24 (72.7)	.37
No	32 (31.1)	23 (32.9)	9 (27.3)	

Data are presented as mean ± standard deviation (SD) or as numbers (percentage). ADL = activities of daily living, BMI = body mass index, CC = calf circumference, GLIM = Global Leadership Initiative on Malnutrition, MNA-SF = Responses to the Mini Nutritional Assessment Scale-Short Form.

Polypharmacy, neuropsychological problems, and psychological stress were found to have an independent relationship with malnutrition after adjusting for age, sex, and other confounding factors (Table 2). Polypharmacy and mild dementia were found to be independently related to ADL disability, but neither of these factors were found to be related to loss of appetite. The only factor found to be independently related to loss of appetite was the loss of pleasure of eating.

**Table 2:** Multivariable logistic regression analysis for the presence of malnutrition, ADL disability, and loss of appetite.

	Adjusted odds ratio	95% confidence interval	*P*
Malnutrition			
Age, years	0.992	0.932–1.057	.81
Female sex	0.862	0.298–2.495	.78
Protein foods, ≥3 per day	0.979	0.333–2.874	.97
Polypharmacy	4.626	1.523–14.056	<.01
Neuropsychological problems	3.487	1.009–12.050	.05
Psychological stress	6.083	2.116–17.050	<.01
Loss of appetite	1.545	0.267–8.948	.63
Loss of pleasure of eating	3.400	0.917–12.605	.07
ADL disability			
Age, years	0.965	1.914–1.019	.20
Female sex	0.742	0.339–2.092	.71
Protein foods, ≥3 per day	1.977	0.785–4.977	.15
Malnutrition	0.954	0.313–2.906	.93
Polypharmacy	2.769	1.070–7.170	.04
Mild dementia	5.922	2.012–17.432	<.01
Psychological stress	1.343	0.471–3.849	.58
Loss of appetite	1.117	0.213–5.849	.90
Loss of pleasure of eating	2.563	0.678–9.692	.17
Loss of appetite			
Age, years	1.121	0.9911–1.269	.07
Female sex	0.992	0.201–4.888	.99
Protein foods, ≥3 per day	0.552	0.103–2.958	.49
Malnutrition	1.219	0.191–7.777	.83
Polypharmacy	0.899	0.167–4.851	.90
Psychological stress	0.364	0.047–2.833	.34
Loss of pleasure of eating	11.996	1.907–75.447	<.01
Eating together at least once per day	0.573	0.094–3.509	.55

ADL = activities of daily living.

Table 3 summarizes the results of the multiple logistic regression analysis performed for drug intake status, with significant differences between the presence of malnutrition and ADL disability after adjusting for age, sex, and other confounding factors. Separate analyses for each type of drug indicated that antihypertensive drugs, PPIs, and anticonstipation drugs were associated with malnutrition, whereas PPIs, anticonstipation drugs, antidyslipidemia drugs, and antidiabetic drugs were associated with ADL disability.

**Table 3:** Multivariable logistic regression analysis for the presence of malnutrition and poor activities of daily living in older individuals with disabilities according to the type of drugs used.

	GLIM criteria^∗^				
Malnutrition^†^	Well-nourished	Malnourished	Adjusted odds ratio	95% confidence interval	*P*
Antihypertensive drugs	27 (38.6)	20 (60.6)	3.297	1.153–9.428	.03
Proton pump inhibitors	19 (27.1)	17 (51.5)	3.885	1.365–11.064	.01
Anticonstipation drugs	17 (24.3)	15 (45.5)	3.094	1.089–8.789	.03
Antiplatelet drugs	14 (20.0)	10 (30.3)	1.523	0.501–4.635	.46
Antidyslipidemia drugs	14 (20.0)	9 (27.3)	1.124	0.369–3.426	.84
Sleeping aids	12 (17.1)	8 (24.2)	1.856	0.561–6.133	.31
Antidiabetic drugs	11 (15.7)	5 (15.2)	0.957	0.242–3.783	.95
Anticoagulants	4 (5.7)	6 (18.2)	3.507	0.724–16.982	.12
Probiotics	4 (5.7)	6 (18.2)	4.596	0.737–28.821	.10
Gastrointestinal motility regulators	3 (4.3)	4 (12.1)	3.911	0.568–26.915	.17

	Bedridden level^∗^				
ADL disability^‡^	Independent	ADL disability			
Antihypertensive drugs	22 (38.6)	25 (54.3)	2.299	0.893–5.917	.09
Proton pump inhibitors	15 (26.3)	21 (45.7)	2.893	1.098–7.622	.03
Anticonstipation drugs	11 (19.3)	21 (45.7)	2.974	1.121–7.918	.03
Antiplatelet drugs	10 (17.5)	14 (30.4)	2.438	0.863–6.887	.09
Antidyslipidemia drugs	8 (14.0)	15 (32.6)	2.992	1.018–8.796	.05
Sleeping aids	10 (17.5)	10 (21.7)	1.293	0.424–3.947	.65
Antidiabetic drugs	6 (10.5)	10 (21.7)	3.651	1.017–13.106	.05
Anticoagulants	4 (7.0)	6 (13.0)	1.552	0.357–6.754	.56
Probiotics	3 (5.3)	4 (8.7)	2.789	0.478–16.260	.25
Gastrointestinal motility regulators	4 (7.0)	3 (6.5)	0.832	0.144–4.804	.84

Data are presented as numbers (percentage). ADL = activity of daily living, GLIM = Global Leadership Initiative on Malnutrition.

∗ The number of participants who used drugs were indicated by n (%). Participants can use more than 1 drug; thus, the percentages do not sum to 100%.

† Odds ratio adjusted for age (continuous), sex (male = 0, female = 1), protein foods per day (3 or more = 0, less than 3 = 1), polypharmacy (less than 5 = 0, 5 or more = 1), psychological stress (no = 1, yes = 1), loss of appetite (no = 0, yes = 1), and loss of pleasure of eating (no = 0, yes = 1).

‡ Odds ratio adjusted for age (continuous), sex (male = 0, female = 1), protein foods per day (3 or more = 0, less than 3 = 1), polypharmacy (less than 5 = 0, 5 or more = 1), rating of dementia (independent = 0, mild dementia = 1), psychological stress (no = 1, yes = 1), loss of appetite (no = 0, yes = 1), and loss of pleasure of eating (no = 0, yes = 1).

**Figure 2:**
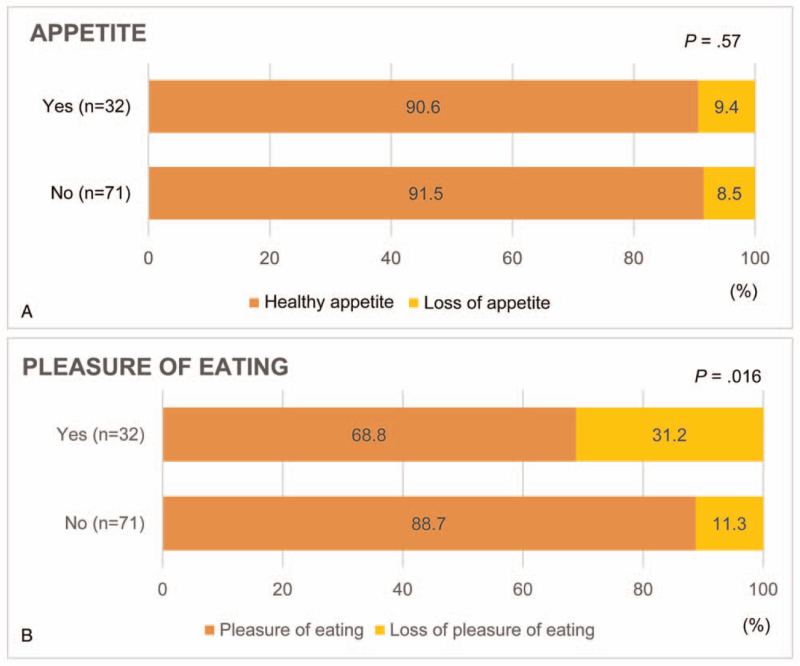
Effect of psychological stress on appetite (A) and pleasure of eating (B).

Figure 2 shows the effect of psychological stress on appetite (Fig. 2A) and pleasure of eating (Fig. 2B). Psychological stress was not found to be related to the loss of appetite; however, it was found to be related to the loss of pleasure of eating.

## 4 Discussion

To the best of our knowledge, this study is the first to assess the effect of polypharmacy on malnutrition and ADL disability among daycare facility users in Japan. Two important conclusions can be drawn from these findings: First, polypharmacy was found to be related to malnutrition and ADL disability among the study participants. The investigation of specific drugs revealed that PPIs were associated with the presence of malnutrition and ADL disability. Second, loss of appetite was found to be related to the loss of pleasure of eating, which in turn was related to psychological stress.

Polypharmacy was significantly associated with malnutrition, even after adjusting for confounding factors. Malnutrition and polypharmacy have an interactive relationship, where malnutrition adversely affects nutritional status and increases the requirement for higher drug doses, thus creating a vicious cycle.^[24]^ Furthermore, polypharmacy and the presence of mild dementia were independently associated with ADL disability. It is known that polypharmacy and potentially inappropriate pharmacotherapy may lead to ADL disability in older individuals who require rehabilitation.^[24]^ A previous study reported that ADL disability uniquely contributes to the risk of incident dementia.^[33]^ We also showed that ADL disability and mild dementia are closely related.

PPI use was independently associated with malnutrition and ADL disability. PPIs are highly effective inhibitors of gastric acid secretion by parietal cells.^[20,34]^ Nutritional evidence suggests that PPIs impair protein absorption and assimilation.^[35]^ Although PPIs have been used to treat gastroesophageal reflux disease, it was recently reported that the long-term use of PPIs increases the risk of sustaining bone fractures and developing *Clostridium difficile* infection.^[36,37]^ Therefore, the Guidelines for the Safe Pharmacotherapy of the Elderly strongly recommend that “PPIs should not be administered for >8 weeks and according to the recommended dose.”^[19]^ In our study, participants in the malnourished group consumed a considerable amount of dietary protein. Thus, it is likely that the impairment of protein absorption and suppression of protein assimilation resulting from PPI use cause malnutrition and ADL disability.

The use of antihypertensive drugs was independently associated with malnutrition. Diuretics used as antihypertensive drugs reduce blood volume, which results in reduced saliva production and the consequent onset of dry mouth.^[38]^ This has been linked to the loss of appetite.^[39]^ In addition, several antihypertensive drugs have a zinc-chelating effect, which may cause hypozincemia. Insufficient amounts of zinc in the body can cause various physical problems, including dysgeusia, loss of appetite, and anemia.^[40]^ Thus, the long-term use of antihypertensive drugs may induce malnutrition.

In this study, there was an independent association between malnutrition and ADL disability among individuals using anticonstipation drugs. The causes of constipation include low food intake, insufficient exercise, and the use of drugs that cause constipation or suppress gastrointestinal motility.^[41]^ We assumed that the participants experienced a loss of appetite as a result of low food intake, insufficient exercise, and other factors.

The use of antidiabetic drugs has been associated with ADL disability. Type 2 diabetes mellitus is a risk factor for sarcopenia and age-related decline in skeletal muscle mass and strength.^[42]^ The presence of diabetes is thought to be associated with ADL disability.

The use of antidyslipidemia drugs was found to be independently associated with ADL disability. Statins are currently the most efficient drugs for the treatment of hypercholesterolemia.^[19]^ However, statin alone^[43]^ or in combination with fibrates have been reported to be associated with a risk of rhabdomyolysis.^[19]^ When used for an extended period, these drugs may cause loss of muscle mass and ADL disability.

We found no relationship between malnutrition, ADL disability, and loss of appetite. Furthermore, polypharmacy was found to be unrelated to loss of appetite. Malnutrition among the study participants was related to neuropsychological problems and psychological stress. It caused a loss of pleasure of eating among the study participants, which was linked to the loss of appetite. On the basis of these findings, there is a need to promote meal-related support from a psychological perspective and ensure that older people are able to enjoy their meals.

In the present study, malnutrition was related to neuropsychological problems and psychological stress. Psychological stress caused a loss of enjoyment of eating among the study participants, which was linked to the loss of appetite. Katsas et al^[44]^ reported that malnutrition among community-dwelling older individuals is associated with cognitive decline. Martin et al^[45]^ reported the factors that contribute to low body weight among community-dwelling older individuals, including eating alone, social isolation, and stressors. Meals are a major source of enjoyment in the daily lives of older people. For older people, meals are important over and above the intake of nutrients. It has been advocated that the focus of nutrition for older people should shift from nutritional status to overall diet.^[46]^ Thus, there is a need to promote meal-related support from a psychological perspective and to ensure that older people are able to enjoy their meals.

This study has several limitations. First, it was a cross-sectional study; thus, we were unable to verify cause-and-effect relationships. Second, we analyzed only data obtained from 103 participants at a single facility. Further, of the 103 participants, 95 participants were older individuals, whereas 8 participants were not older individuals. Therefore, selection bias may have occurred. Third, although we examined all the drugs used by the participants, we were unable to investigate the relationship between each drug and loss of appetite. Hence, multicenter prospective studies with large sample sizes are needed to determine the relationship between polypharmacy and malnutrition and ADL disability. Fourth, we did not utilize verified screening tools, such as the simple nutritional assessment questionnaire (SNAQ),^[47]^ to identify older individuals who were at risk of anorexia. Thus, there remains a need to investigate the relationship between malnutrition and anorexia using screening tools such as the SNAQ. In addition, our diet-related survey assessed only the frequency of meals. There remains a need to examine this issue using a quantitative meal survey. Finally, only a few participants underwent regular body weight measurements. Thus, we were unable to accurately determine the rate of body weight loss; therefore, we may have underestimated the malnutrition status.

Nevertheless, the prevalence of malnutrition and ADL disability among the daycare facility users we investigated was similar to that among other daycare facility users.^[8,9]^ Therefore, the results of this study may be applied to other daycare facility users.

In Japan, initiatives designed to resolve the problem of polypharmacy have already been initiated. The 2016 revision of the medical payment system provided assistance funding for cases in which the number of drugs prescribed was reduced. The 2020 revision of the medical payment system led to pharmacies being able to centrally ascertain patients’ drug data, which allows them to identify whether a patient is taking duplicate medications. In relation to this, assistance funding is provided to pharmacies that make suggestions to physicians for the reduction and elimination of duplicate medications.^[48]^

The Ministry of Health, Labour, and Welfare's Report on Public Health Administration and Services indicated that the total number of dispensing pharmacies throughout Japan in 2018 was 59,613.^[49]^ Between 10,000 and 15,000 of these are dispensing pharmacies that are targeted for registration by 2025 as health support pharmacies that will be actively engaged in comprehensive community care.^[50]^ Health support pharmacies function as “primary care pharmacies” in addition to their conventional pharmacy services; thus, they are expected to play a role in community resident health support through interdisciplinary cooperation and collaboration with related organizations.

## Acknowledgments

The authors wish to express their deep appreciation to all the users and staff at Assist Reha Yono and Amano Nobuko at Konan Women's University for their cooperation in this study. The authors would also like to express their deep appreciation to Iwasaki Eiji, President and CEO of Medical Assist Co., Ltd.; Nakayama Kenichi, Director of Assist Reha Yono; and nurse Thou Asako for their understanding and cooperation.

## Author contributions

**Conceptualization:** Tomiyo Nakamura, Takashi Itoh, Shoko Imai, Hidenori Kominato.

**Data curation:** Tomiyo Nakamura, Takashi Itoh, Aiko Yabe, Shoko Imai.

**Formal analysis:** Tomiyo Nakamura.

**Funding acquisition:** Hidenori Kominato.

**Investigation:** Takashi Itoh, Aiko Yabe, Shoko Imai, Yoshimi Nakamura, Yasuko Mizokami, Yuki Okouchi, Akito Ikeshita.

**Methodology:** Tomiyo Nakamura, Takashi Itoh.

**Project administration:** Tomiyo Nakamura, Takashi Itoh, Hidenori Kominato.

**Resources:** Hidenori Kominato.

**Software:** Tomiyo Nakamura.

**Supervision:** Tomiyo Nakamura, Hidenori Kominato.

**Validation:** Tomiyo Nakamura.

**Visualization:** Tomiyo Nakamura.

**Writing – original draft:** Tomiyo Nakamura.

**Writing – review & editing:** Takashi Itoh, Aiko Yabe, Shoko Imai, Yoshimi Nakamura, Yasuko Mizokami, Yuki Okouchi, Akito Ikeshita, Hidenori Kominato.
